# Does the SARS-CoV-2 spike really have an Achilles heel?

**DOI:** 10.1172/JCI168080

**Published:** 2023-04-17

**Authors:** Shiv Pillai

**Affiliations:** Ragon Institute of MGH, MIT, and Harvard, Cambridge, Massachusetts, USA.

## Abstract

The continued emergence of SARS-CoV-2 variants and waning vaccine immunity are some of the factors that drive the continuing search for more effective treatment and prevention options for COVID-19. In this issue of the *JCI*, Changrob, et al. describe an anti-SARS-CoV-2 spike antibody, isolated from a patient, that targets a vulnerable site on the spike protein receptor binding domain when it adopts a configuration called the “up” conformation. This antibody cross-neutralized all variants studied, including recent Omicron subvariants, and was protective against multiple variants in a hamster model. These results are of interest when considering the next generation of prophylactic and therapeutic antibodies for COVID-19, but they may also shape future approaches to vaccination against SARS-CoV-2.

## SARS-CoV and antigenic variation

The original SARS virus, now called SARS-CoV-1, emerged in China in the winter of 2002–03 and was transmitted to at least 28 countries across the world before it was extinguished in the summer of 2003 (1). In the nine months between the onset and disappearance of this disease, the total number of cases established worldwide was 8,437, and the number of deaths reported was 813. SARS was a devastating disease, and although the first officially described cases of SARS and the first cases of severe COVID-19 were temporally separated by seventeen years, the two diseases were clinically indistinguishable when they were first seen.

By early 2020 it was recognized that SARS-CoV-1 had clearly not been anywhere as transmissible as its close cousin, SARS-CoV-2 (2). By early 2023, SARS-CoV-2 infections had officially topped 750 million, but likely far more people had been infected. For the first six months of the COVID-19 pandemic, concerns about possible pathogenic variants of SARS-CoV-2 largely remained dormant, and, indeed, few emerging variants were initially described. One factor influencing most, and, in retrospect, possibly overly optimistic, thinking at that time was the fact that coronaviruses have a robust proofreading machinery (3). It was, therefore, assumed by many that rapidly mutating variants of this virus were unlikely to pose a major problem. But even with the relative stability of this viral genome, it began to be recognized that, given the sheer size of the pandemic, variants that enhanced viral propagation were bound to be selected for. The first variant of the spike gene that was identified, more than six months into the pandemic, was the D614G variant, which was not altered antigenically but had a propagation advantage (4). However, once immunity in populations at large became more widespread, initially from natural infection and later from vaccination, humoral immune pressure created a major driving force for the further evolution of SARS-CoV-2 (5–7).

## An unusual broadly neutralizing antibody against SARS-CoV-2

Both vaccination and natural infection result in the generation of neutralizing antibodies. Cloning of the activated B cells that make such antibodies in patients led to the recombinant generation and clinical use of many patient-derived monoclonal antibodies, which can be administered either as single antibodies or as cocktails. Many of these monoclonal antibodies were successful in slowing infection and could be used prophylactically, especially in immunodeficient and/or immunosuppressed individuals. However, most of these monoclonal antibodies started to lose efficacy in terms of neutralization following the emergence of Omicron and its subvariants. As a result, many monoclonal antibodies that were recently widely used are currently no longer recommended for SARS-CoV-2 infection and prophylaxis in the United States.

For viruses that have a propensity to evade host immunity in part by generating variant spike and envelope proteins, an all-too-often unrealized goal of vaccination is the generation of broadly neutralizing antibodies that can recognize and protect against all or most variants. Attempts to generate vaccines against HIV and influenza that generate such broadly neutralizing antibodies have so far been unsuccessful. Although current vaccines deployed in the United States against SARS-CoV-2, all based on the expression of the spike protein of the ancestral SARS-CoV-2 strain, are very effective in terms of protection from hospitalization and severe disease, they no longer protect effectively against viral entry by newer antigenically diverse variants, largely because they do not effectively generate broadly neutralizing antibodies. Broadly neutralizing antibodies that can cross-neutralize all known variants and subvariants of SARS-CoV-2, and that can protect from infection by all variants in animal models, have the potential to be used therapeutically and prophylactically.

In this issue of the *JCI*, Changrob and authors may have discovered one such antibody (8). This monoclonal antibody that they refer to as S728-1157 was derived from a single B cell in a recovering patient and was capable of cross-neutralizing all variants tested, including Delta and six different Omicron subvariants. S728-1157 also protected hamsters against in vivo challenge with these variants. Based on an analysis of the structures of a range of neutralizing antibodies bound to the spike protein (9), S728-1157 is categorized as a Class 1 antibody — a category of antibodies that neutralize by binding to the receptor binding domain (RBD) when it presents in the up conformation. In many SARS-Cov-2 vaccines, including all used in the United States, the spike protein is stabilized in the down conformation, also known as prefusion state, by the introduction of two prolines (2P) into the S2 domain (10), and, in these trimers, one RBD presents in the up state. A hexaproline (6P) stabilized form of Spike, not yet deployed in human vaccination, ensures that two RBDs are in the up conformation (11), and was utilized in many of the studies by Changrob, et al. (8).

**Figure 1:**
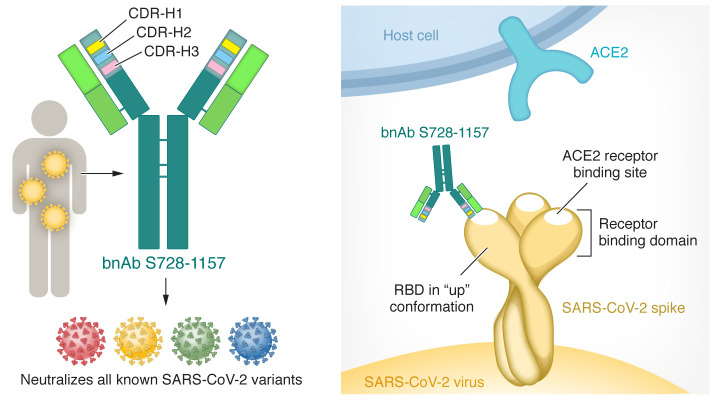
The broadly neutralizing S728-1157 antibody can neutralize all known SARS-CoV-2 variants and subvariants. The S728-1157 antibody binds to a site of the Spike RBD when it is in the up conformation. When the Spike RBD is in the up conformation, the RBS is able to make contact with ACE2 on host cells to mediate viral entry. The S728-1157 antibody requires the invariant CDR-H1 and CDR-H2 loops, encoded by *IGHV3-66*, for binding. It also extensively utilizes the unique CDR-H3 loop in an unmutated form.

The site where S728-1157 binds to the spike RBD when it is in the up conformation corresponds to the patch — also known as the receptor binding site (RBS) — required for ACE2 binding (Figure 1), and the specific epitope it binds to is referred to as the RBS-A epitope. Indeed, 6 distinct epitopes recognized by different antibodies have been previously categorized in the SARS-CoV-2 spike RBS. Most class 1 antibodies utilize the invariant CDR-H1 and CDR-H2 loops found in two human heavy-chain-variable (VH) genes, *IGHV3-53* and *IGHV3-66*, and, indeed, S728-1157 utilizes the CDR-H1 and CDR-H2 loops of *IGHV3-66*. However, S728-1157 is atypical in that it also utilizes the side chains of a large number of amino acids in the largely unmutated CDR-H3 loop of this antibody to make numerous contacts with the RBS. CDR-H1 and CDR-H2 are inherited (in the germline variable [V] gene segment). However, CDR-H3 is randomly generated by the recombination of the V, diversity (D), and joining (J) gene segments during B cell development and includes D and J segments and junctional residues. The authors assumed that because multiple residues on the RBS form contacts with the CDR-H3 of S728-1157, fairly extensive hydrogen bonding and hydrophobic interactions between this antibody and the spike RBD may persist despite mutation of the RBD in variant viruses. These constraints may explain the antibody’s effectiveness in terms of broad neutralization.

It should be noted that other groups have identified antibodies that were also seen to be broadly neutralizing, including some that also have been tested against Omicron subvariants, but most have not been tested as extensively as S728-1157 for in vivo efficacy (12, 13).

## Determining whether vaccination will achieve broad neutralization

The S728-1157 antibody is well suited for therapeutic and prophylactic use. But is knowledge about its existence relevant for vaccination strategies?

One metric that we use to test the efficacy of currently used vaccines against SARS-CoV-2 is protection from severe respiratory illness and hospitalization, and, by this measure, vaccines have been extraordinarily successful (14). Currently the only established rigorous in vitro correlate of vaccinal protection against SARS CoV-2 is neutralization (15, 16). Vaccines still protect robustly, but likely largely via cytotoxic T cells, antibody dependent cellular cytotoxicity (ADCC), and phagocyte-mediated internalization of antibody-coated viruses. However, there are still a number of unanswered questions about all current parenterally administered SARS-CoV-2 vaccines. The durability of vaccinal immune responses still requires investigation; vaccines in use in the United States generate systemic immunity but are less focused on the upper respiratory tract. It still needs to be established if absolute protection from infection can be achieved by broadly neutralizing antibodies, and, if these antibodies need to be in larger concentrations at or close to the site of entry of the virus to be most effective.

Changrob and colleagues showed that the S728-1157 antibody (as well as many other neutralizing antibodies) more readily recognized the 6P modified artificially generated prefusion hexaproline form of the spike protein, which results in two RBDs in a spike molecule presenting in the up conformation (8). In order for natural infection to occur, the RBD must be in the up conformation at the time of viral entry and that is likely how S728-1157 B cells were selected and activated in the patient they were derived from. It is, however, clear that the frequency of naive and memory B cells with germline heavy chain VDJ exons that resemble the one identified in S728-1157 are likely to be very low. There are, therefore, a few issues that would need to be kept in mind if attempts are made to vaccinate humans with a hexaproline spike protein. Apart from the phenomenon referred to as “original antigenic sin,” wherein preexisting memory B cells are favored with every subsequent cross-reactive antigenic exposure, there will also be a low precursor frequency of the appropriate naive and memory B cells that can initiate a new broadly neutralizing response. So, generating effective, broadly neutralizing, antibody responses in a large swath of the population will likely be difficult.

Broadly neutralizing antibodies target sites of vulnerability on a virus that are shared by all variants. But will such sites really be preserved in SARS-CoV-2? This malleable virus is constantly evolving and can mutate so-called “essential” sites at the Spike-ACE2 interface to evade humoral immunity and then make compensatory mutations that then permit ACE2 binding (17). Would an antibody such as S728-1157, that is broadly neutralizing but utilizes numerous sites of contact with a vulnerable site on the Spike protein, have a greater chance of not being evaded? In other words, could a patch on the ACE-2 binding RBS really prove to be an Achilles heel for SARS-CoV-2?

Even if the wide generation in a large population of a seemingly ideal, sterilizing, broadly neutralizing antibody response targeting this particular site of vulnerability were to be achieved, infection might still occur in some individuals, possibly those with an immunodeficiency, thus triggering further viral mutations. It is, therefore, fairly likely that some combination of immune-evasive and compensatory mutations in the virus might eventually contribute to the emergence of new SARS-CoV-2 subvariants, even if effective broadly neutralizing humoral immunity against all substrains that are circulating at a particular point of time could be widely achieved in the population at large in the future. Is it too much to hope that our immune systems can be modulated to generate many S728-1127–like antibodies and that, like Paris’s mythical arrow, the antibodies targeting this Achilles heel will prevail?

## Version 1 04/17/2023

Electronic publication
